# Differential Expression and Localization of Branchial AQP1 and AQP3 in Japanese Medaka (*Oryzias latipes*)

**DOI:** 10.3390/cells8050422

**Published:** 2019-05-08

**Authors:** Laura V. Ellis, Rebecca J. Bollinger, Hannah M. Weber, Steffen S. Madsen, Christian K. Tipsmark

**Affiliations:** 1 Department of Biological Sciences, J. William Fulbright College of Arts and Sciences, University of Arkansas, Fayetteville, AR 72701, USA; lvellis@email.uark.edu (L.V.E.); rebeccajbollinger@gmail.com (R.J.B.); hweber@email.uark.edu (H.M.W.); 2 Department of Biology, Natural Science Faculty, University of Southern Denmark, 5230 Odense, Denmark; steffen@biology.sdu.dk

**Keywords:** gill, aquaporin, prolactin, cortisol, osmotic stress, salinity, cell volume regulation

## Abstract

Aquaporins (AQPs) facilitate transmembrane water and solute transport, and in addition to contributing to transepithelial water transport, they safeguard cell volume homeostasis. This study examined the expression and localization of AQP1 and AQP3 in the gills of Japanese medaka (*Oryzias latipes)* in response to osmotic challenges and osmoregulatory hormones, cortisol, and prolactin (PRL). *AQP3* mRNA was inversely regulated in response to salinity with high levels in ion-poor water (IPW), intermediate levels in freshwater (FW), and low levels in seawater (SW). AQP3 protein levels decreased upon SW acclimation. By comparison, AQP1 expression was unaffected by salinity. In ex vivo gill incubation experiments, *AQP3* mRNA was stimulated by PRL in a time- and dose-dependent manner but was unaffected by cortisol. In contrast, *AQP1* was unaffected by both PRL and cortisol. Confocal microscopy revealed that AQP3 was abundant in the periphery of gill filament epithelial cells and co-localized at low intensity with Na^+^,K^+^-ATPase in ionocytes. AQP1 was present at a very low intensity in most filament epithelial cells and red blood cells. No epithelial cells in the gill lamellae showed immunoreactivity to AQP3 or AQP1. We suggest that both AQPs contribute to cellular volume regulation in the gill epithelium and that AQP3 is particularly important under hypo-osmotic conditions, while expression of AQP1 is constitutive.

## 1. Introduction

Euryhaline fish, such as the Japanese medaka (*Oryzias latipes*), are capable of acclimating to both freshwater (FW) and seawater (SW) environments. In FW, they are challenged by osmotic water inflow and passive ion loss and must combat this by actively absorbing ions from the environment and getting rid of excess water [1,2]. Regulation of the body’s ion balance is primarily taken care of by the gill, and water balance is maintained by renal production of copious amounts of hypo-tonic urine [2]. In SW, integumental ion and water fluxes are basically reversed, meaning that the passive influx of ions and loss of water must be compensated for by the active secretion of monovalent ions in the gill and solute-linked water absorption in the gastrointestinal tract. Thus, the function of the gill has extreme plasticity in euryhaline fish. Ionocytes in the gill are the cells responsible for the active transport of ions in both directions depending on the salinity. In addition to a high density of basolateral Na^+^,K^+^-ATPase (Nka), ionocytes are equipped with specific apical and basolateral ion transport proteins that work together in ion absorption and excretion. One FW-type ionocyte specifically expresses a gill-specific paralog of apical sodium chloride cotransporter protein (Ncc2: [3]), and SW ionocytes express basolateral sodium-potassium-2-chloride cotransporter [4] and an apical cystic fibrosis transmembrane conductance regulator protein [5]. In addition to modifications in the expression pattern of transmembrane ion transport proteins, junctional complexes between these cells are modified from having a tight nature in FW to prevent sodium ion loss to being leakier in SW to allow for the removal of excess sodium ions [6].

The gill is a complex three-dimensional structure consisting of primary filaments and secondary lamellae, which create an expansion of the surface area in direct contact with the surrounding water [2]. By having a very thin structure, the gill epithelium is optimized for gas transport but also becomes a gate for passive exchange of water and ions between the fish and its environment (the "osmo-respiratory compromise" [7]). Ideally, branchial transepithelial water permeability should be kept to a minimum; however, the high gas permeability puts a particular strain on epithelial cells covering the surface of the gill in terms of maintaining osmotic and cell volume homeostasis. Regulation of the osmotic permeability of the branchial epithelium therefore becomes an interesting topic, which has been scarcely investigated thus far. Transepithelial water permeability (osmotic as well as diffusional) of gills has been analyzed in a few studies and has been found to be highest in FW and lowest in SW (e.g., eel [8,9]).

Cell membranes are generally quite permeable to water [10]. Their permeability may be increased by the presence of aquaporins (AQPs), major intrinsic proteins that form transmembrane channels facilitating the transport of water and certain small solutes [11,12,13,14]. Functionally, aquaporins facilitate transepithelial movement of water, as well as cell volume regulation [14,15,16,17,18]. Mammals have 13 AQP paralogs, the orthodox AQPs (AQP0, -1, -2, -4, -5, -6, and -8), the aquaglyceroporins (Aqp3, -7, -9, and -10), and the super-AQPs (AQP11 and -12) [12,15,16]. Due to multiple genomic duplication events, certain teleost fishes such as Atlantic salmon (*Salmo salar*), have up to 42 AQP paralogs with multiple forms of the same AQP [19]. In contrast, Japanese medaka express 11 paralogs. AQP1 and AQP3 are the main paralogs expressed in the gills of different fish species and are speculated to play roles in the regulation of branchial water homeostasis [20]. With the gill being in direct contact with a surrounding medium that typically has a very different osmotic composition compared with the internal milieu, it would be detrimental if AQPs created transcellular pathways for water movement in the superficial epithelial cell layer. Thus, it would rather be expected that they are localized deeper in the epithelium and/or in an asymmetrical pattern and that their role is concerned with cell volume regulation. However, due to the lack of knowledge in this subject, studies are necessary to investigate their localization and expression dynamics in response to salinity.

When establishing aquaporin expression dynamics, it is interesting to also investigate the role of the endocrine system in the regulation of aquaporins. Several studies have addressed the endocrine regulation of branchial ion transport mechanisms [21], whereas we know relatively little about the expressional regulation of AQPs in any tissue of teleosts. Prolactin (PRL) is widely accepted as a FW adapting hormone in teleosts that stimulates ion uptake and the associated molecular elements in FW-type ionocytes and, at the same time, reduces the abundance of secretory SW-type ionocytes [22,23,24]. Elevated plasma PRL may reduce circulating insulin-like growth factor-1 (IGF-1), which in turn leads to decreased branchial salt secretion and differentially regulates Nka alpha-subunit isoforms [21,24,25,26]. In Mozambique tilapia (*Oreochromis mossambiccus*), PRL increased branchial *AQP3* expression, while cortisol had the ability to block the stimulation of *AQP3* [27]. Dose-dependent stimulation of *NCC2* by PRL has been shown in zebrafish (*Danio rerio*) and medaka, and expression of this gene may be used as a marker for select FW-type ionocytes [28,29]. Cortisol is a mediator of both FW- and SW-adapting effects in the gill. It may interact with growth hormone (Gh) and IGF-1, thereby promoting salt secretory mechanisms and SW-acclimation [24,30,31]. On the other hand, cortisol can also interact with PRL and either exacerbate or antagonize the ion-regulatory effects of PRL [26,27]. Previous work in various fish species does not provide a clear consensus concerning salinity effects on AQP1 and AQP3 nor concerning their cellular localization within the gill [20]. Therefore, we here used the euryhaline medaka model to advance our understanding of these AQP paralogs’ significance to gill function.

Japanese medaka has previously been used as a model organism for studying ionic and osmotic regulation in a euryhaline teleost [3,29,32,33,34,35], and aquaporin tissue distribution was characterized in a previous study with a focus on the dynamics in the intestine [33]. The current study aims to answer three main questions: (i) in which cell types do we find the two aquaporin paralogs, AQP1 and AQP3, expressed in the gill of medaka?; (ii) how are branchial AQP1 and AQP3 mRNA and protein expression and localization affected by environmental salinity?; and (iii) are PRL and cortisol involved in endocrine control of AQP expression? Time-course salinity transfer experiments from FW to ion-poor water and from FW to SW were performed to investigate the expression dynamics at the mRNA and protein level. This was combined with confocal immunofluorescence analyses of branchial localization of the two paralogs, and ex vivo experiments were used to investigate the role of cortisol and PRL in regulating AQP1 and AQP3 expression in the gill.

## 2. Materials and Methods

### 2.1. Fish and Maintenance

Adult Japanese medaka (*O. latipes*, Temmink and Schlegel; total length: 25–35 mm, weight range: 250–350 mg) were obtained from Aquatic Research Organisms, Inc. (Hampton, NH, USA; N = 118). Fish were acclimated and kept in recirculated de-chlorinated biofiltered fresh water (FW; in mmol L^−1^: 0.34 Na^+^, 0.64 Ca^2+^, 0.09 Mg^2+^, 0.03 K^+^). Medaka were maintained in a 14 h light/10 h dark photoperiod at a temperature of 20 °C. They were fed daily with Tetramin tropical flakes (Tetra, United Pet Group, Blacksburg, VA, USA), and food was withheld 24 h prior to any sampling. Upon sampling, the fish were sacrificed by cervical dislocation. Subsequent pithing of the brain was performed, and the gill apparatus was removed. All handling and experimental procedures were approved by the Animal Care and Use Committee of the University of Arkansas (IACUC 14042 and 17091).

### 2.2. Salinity Transfer Experiments: FW–SW, FW–Ion-Poor Water (IPW).

To investigate the response of medaka to hyper-osmotic environments, 10 female and 10 male FW-acclimated medaka were transferred to both sham FW conditions and SW (28 ppt; Instant Ocean, Spectrum Brands, Blacksburg, VA, USA; N = 40) and sampled after 6, 24, and 168 h (n = 6 per group). The gills were removed and washed with phosphate-buffered saline (PBS, Roche Diagnostics, Indianapolis, IN, USA), and then, 4 gill arches were placed directly into 250 μL of TRI Reagent^®^ (Sigma Aldrich, St. Louis, MO, USA) for mRNA analyses, while the other four were placed directly into 1.5 mL sonication tubes in 1X lithium dodecyl sulfate (LDS) NuPAGE Sample Buffer (Thermo Fisher Scientific, Waltham, MA, USA) with 50 mmol L^−1^ dithiothreitol (DTT, GE Healthcare Bio-Sciences, Pittsburgh, PA, USA). 

To test the response of medaka to a dilute hypo-osmotic environment, 10 female and 10 male FW-acclimated medaka were transferred to sham FW conditions and IPW (90% deionized water and 10% tap water; N = 40). After 6, 24, and 168 h, gills (n=6 per group) were sampled and treated as described above.

### 2.3. Ex vivo Hormone Incubation Experiments

For all ex vivo incubation experiments, gill explants from FW-acclimated medaka (N = 38) were dissected, and the 4 paired gill arches with cartilage intact were separated from one another, washed with PBS, and placed for 1 h of pre-incubation in Dulbecco’s modified Eagle’s medium (DMEM; Cellgro by Corning, manufactured by Mediatech, Inc., Manassas, VA, USA) with the addition of 50 U mL^−1^ of penicillin and 50 µg mL^−1^ of streptomycin (Thermo Fisher Scientific, Waltham, MA, USA), as previously described by Bossus et al. [29]. Four separate experiments were conducted: Cortisol dose–response (0, 0.1, 1, and 10 μg of cortisol mL^−1^) with 18 h incubation time (n = 5–6 per group); PRL dose–response (0, 0.01, 0.1, and 1μg of ovine PRL mL^−1^) with 18 h incubation time (n = 7–8 per group); PRL time-course experiment using a dose of 1μg mL^−1^ and 2, 6, and 18 h of incubation (n = 7–8 per group); and a combination experiment with a control, cortisol 10 μg mL^−1^, PRL 1μg mL^−1^, and cortisol 10 μg mL^−1^ + PRL 1μg mL^−1^ (n = 7–8 per group). Cortisol (hydrocortisone hemisuccinate sodium salt; Sigma-Aldrich, St. Louis, MO, USA) was dissolved in molecular biology water-ultra pure (Sigma Aldrich, St. Louis, MO, USA). Purified ovine PRL (AFP10692C) was obtained from the National Hormone and Peptide Program (Torrance, CA, USA) and dissolved in molecular biology water-ultra pure (Sigma Aldrich, St. Louis, MO, USA). Hormone experiments were conducted by randomizing gill arches from pre-incubation and assigning 2 gill arches to each treatment (each fish provided gill tissue to all treatments). The experiments were terminated by transferring gill explants directly into 250 μL of TRI Reagent^®^ (Sigma Aldrich, St. Louis, MO, USA) for RNA isolation.

### 2.4. RNA Isolation, cDNA Synthesis, and Real-Time qPCR

RNA isolation was conducted according to the manufacturer’s protocol (TRI Reagent^®^; Sigma Aldrich, St. Louis, MO, USA). All samples were homogenized using a Power Max 200 rotating knife homogenizer (Advanced Homogenizing System; Manufactured by PRO Scientific for Henry Troemner LLC, Thorofare, NJ, USA). In individual experiments, 300 or 500 ng of total RNA was used for cDNA synthesis using the Applied Biosystems High Capacity cDNA Reverse Transcription kit (Thermo Fisher). qPCR primers were previously validated and published in Bossus et al. [34] and Madsen et al. [33]. Elongation Factor 1 alpha (*EF1a*), beta actin (*bact*), and ribosomal protein L7 (*RPL7*) were analyzed as normalization genes in all experiments. Real-time qPCR was run on a Bio-Rad CFX96 platform (BioRad, Hercules, CA, USA) using SYBR^®^ Green JumpStart™ (Sigma Aldrich, St. Louis, MO, USA). qPCR cycling was conducted using the following protocol: initial denaturation/activation phase (94 °C) for 3 min, 40 cycles of a 15 s denaturation step and an annealing/elongation step for 60 s (60 °C), followed by a melting curve analysis at an interval of 5 s per degree from 55 to 94 °C. The absence of primer–dimer association was verified with no template controls (NTC). As an alternative to DNAse treatment, the absence of significant genomic DNA amplification was confirmed using total RNA samples instead of cDNA in a no reverse transcriptase control (NRT). Primer amplification efficiency was analyzed using a standard curve method with dilutions of the primers from 2 to 16 times. Amplification efficiency was used to calculate the relative copy numbers of the individual targets. Relative copy numbers were calculated by *E*_a_^ΔCt^, where *C*_t_ is the threshold cycle number and *E*_a_ is the amplification efficiency [36]. Data were normalized to the geometric mean of the three normalization genes and were presented relative to the control group.

### 2.5. Western Blot Analysis

Medaka gills from the salinity transfer experiments were placed directly into 1.5 mL sonication tubes in 1x LDS buffer with 50 mM DTT and then placed in an ultrasonic bath (Ultrasonic Liquid Processor 3000, Farmingdale, NY, USA). Gill samples were sonicated and manually homogenized in accordance with the procedure described by Bollinger et al. [37]. Following sonication, samples were transferred to microcentrifuge tubes and heated at 70 °C for 10 min. Denatured protein samples were run on a 4%–12% Bis-Tris Gel with 2-(*N*-morpholino)ethanesulfonic acid sodium dodecyl sulfate (MES SDS) running buffer with antioxidants. Gels were electrophoresed and then blotted onto 0.2 µm nitrocellulose membranes with transfer buffer containing 10% methanol. Membranes were blocked with LI-COR Blocking Buffer (LI-COR Biosciences, Lincoln, NE, USA) for 1 h at room temperature. Membranes were incubated with a cocktail of affinity-purified homologous rabbit anti medaka AQP1 antibody validated previously [33] or rabbit anti medaka AQP3 antibody (see below), in combination with mouse anti human β-actin (Abcam; cat. no. ab8224) in blocking buffer and incubated overnight at 4 °C on an orbital rotator. Antibodies were used at the following concentrations: AQP1 (0.1 µg mL^−1^), AQP3 (0.1 µg mL^−1^), β-actin (0.2 µg mL^−1^). Membranes were washed four times for 5 min with 1X tris-buffered saline with Tween 20 (TBST: 20 mM Tris, 140 mM NaCl and 0.1% Tween-20). Western blotting solutions and materials were NuPAGE^TM^ from Thermo Fisher, unless stated otherwise. Incubation with secondary antibodies in blocking buffer was performed in dark conditions at room temperature for 1 h (IRDye^®^ 800CW Goat anti-Rabbit IgG and IRDye^®^ 680LT Goat anti-Mouse IgG, LI-COR Biosciences, Lincoln, NE, USA). After washing, membranes were dried and imaged using an Odyssey infrared scanner (LI-COR Bioscience, Lincoln, NE, USA). The AQP3 antibody was generated in rabbit against an epitope in the cytoplasmic C-terminal domain of the medaka AQP3 sequence CFHVEGEVRDKREKM, chosen based on antigenicity and its low similarity with other proteins (GenScript, Piscataway, NJ, USA). The AQP3 sequence has some identity with other predicted proteins in Japanese medaka (https://blast.ncbi.nlm.nih.gov/Blast.cgi; identity is defined as 6–7 of 14 amino acids). These are high-molecular-weight proteins (87, 188, and 212 kDa) that do not occur on western blots. The specificity of the medaka AQP3 was validated by control neutralization with the antigenic peptide in 400x molar excess overnight at 4 °C before probing the membrane. Aquaporin expression (band intensity) was quantified using Image Studio version 2.0 software (LI-COR Biosciences, Lincoln, NE, USA) and normalized against β-actin loading control. The 7-day treatment group was chosen for protein determination to allow for the translation of the messenger and the buildup of the functional protein.

### 2.6. Immunohistochemistry

Gills from medaka transferred from FW to FW, IPW, or SW for 7 days were fixed overnight in 4% phosphate-buffered paraformaldehyde (PFA) and then rinsed and stored in 70% EtOH at 4 °C. They were dehydrated in graded series of EtOH and xylene and embedded in paraffin. Then, 5-μm thick sagittal sections were cut on a microtome, and sections were placed on Superfrost Plus slides (Gerhard Menzel GmbH, Braunschweig, Germany) before being heated for 2 h at 60 °C. The tissue sections were hydrated through washes in xylene; 99%, 96%, and 70% EtOH; and finally, Na-citrate (10 mM Na-citrate, pH 6.0). Antigen retrieval was performed by boiling the sections in the citrate solution for 5 min in a microwave oven and leaving them in the warm citrate solution for 30 min before being washed 2 times for 5 min each in PBS. This was followed by blocking for 1 h (3% bovine serum albumin/2% goat serum in PBS). The sections were then incubated overnight at 4 °C with a cocktail of primary rabbit antibodies against medaka AQP1 (1 µg mL^−1^) or AQP3 (0.8 µg mL^−1^) in combination with a mouse antibody against the alpha subunit of the Nka (a5; the Developmental Studies Hybridoma Bank developed under the auspices of the National Institute of Child Health Development and maintained by the University of Iowa, Department of Biological Sciences, Iowa City, IA, USA) in blocking buffer. The homologous Japanese medaka aquaporin antibodies (AQP1 and AQP3) were the same as those used in the western blot analysis described above. Sections were washed repeatedly in PBS before being incubated with a cocktail of secondary antibodies (goat anti-rabbit IgG Oregon Green^®^ 488/goat anti-mouse IgG Alexa Fluor® 568, 1:1000, Invitrogen, Carlsbad, CA, USA) for 1 h at 37 °C. Sections were finally washed 4 times for 5 min in PBS, and cover slips were mounted using ProLong Gold antifade reagent (Invitrogen). In some cases, nuclei were stained with 4’,6-diamidino-2-phenylindole (DAPI, 0.1 μg mL^−1^ in PBS) for 10 min after washing with PBS and before mounting. Slides were visualized on a Zeiss LSM510 confocal microscope (Carl Zeiss Microscopy GmbH, Jena, Germany), and representative images were collected using the Zeiss Image Browser 4.2.0 software (Jena, Germany) and processed using Image J (National Institutes of Health and the Laboratory for Optical and Computational Instrumentation, LOCI, University of Wisconsin, USA [38]).

### 2.7. Statistical Analysis

All data analysis was conducted using GraphPad Prism 6.0 software (San Diego, CA, USA). Dose-dependent ex vivo experiments were analyzed using one-way ANOVA followed by Tukey’s multiple comparisons test. Data from the salinity transfer experiments, PRL and cortisol time-course experiment, and hormone combination experiment were analyzed via two-way ANOVA followed by Sidak multiple comparisons test of time-matched groups, when there was significant interaction between the two factors (salinity and time or PRL and cortisol). Western blot data were analyzed using two-tailed Student’s t-test. When required, data were log or square-root transformed to meet the ANOVA assumption of homogeneity of variances as tested with Bartlett’s test. Significant differences were accepted when *P* < 0.05.

## 3. Results

### 3.1. Aquaporin mRNA Response to Osmotic Challenges

**Figure 1:**
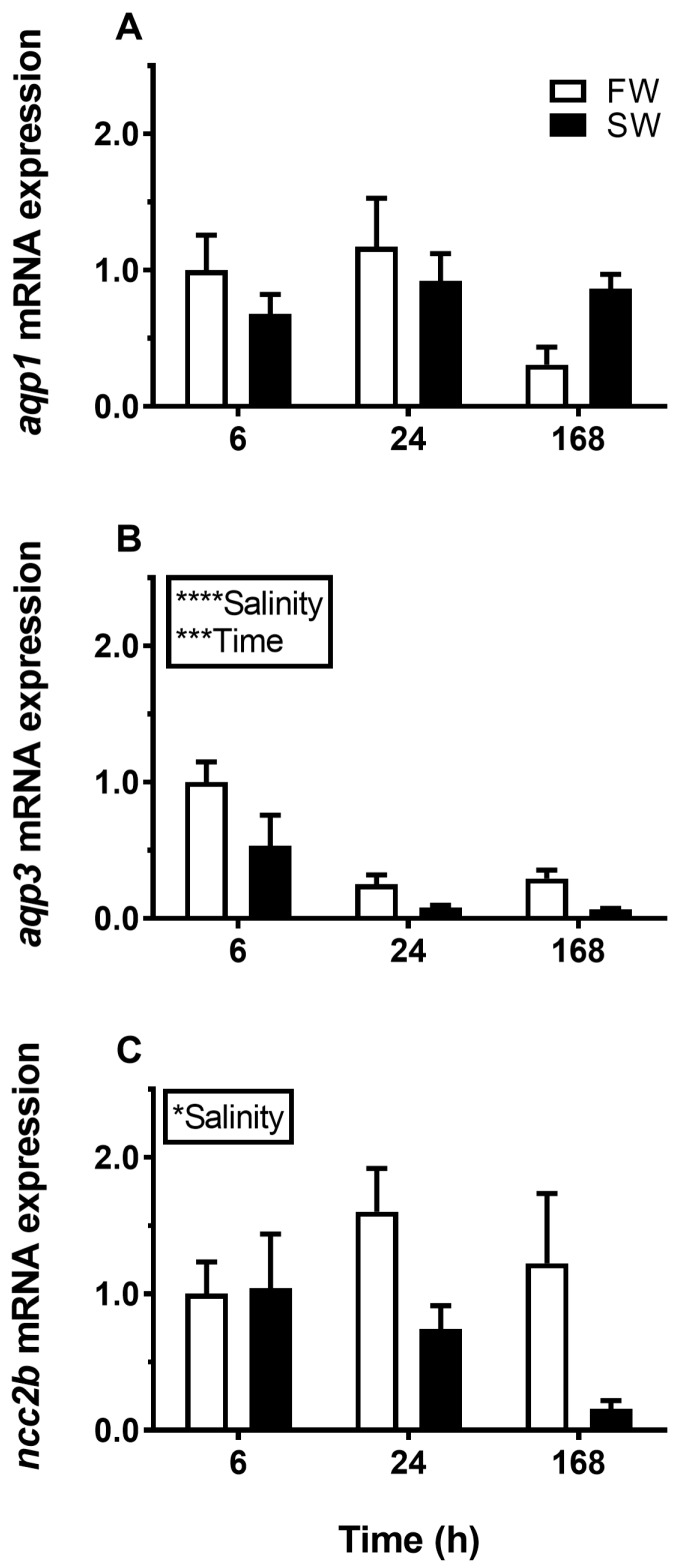
Effect of transfer from freshwater (FW) to (SW) on branchial *aquaporin 1* (*AQP1)* (**A**), *AQP3* (**B**), and *NCC2b* (**C**) expression in medaka at 6, 24, and 168 h timepoints. FW–FW sham transfer (open bars), FW–SW transfer (solid bars). The level of each target was normalized to the geometric mean of the normalization genes *EF1a*, *bact*, and *RPL7* and presented relative to the control group at 6 h. Data are presented as mean ± standard error of the mean (n = 7–8). Statistically significant factor effects (two-way ANOVA, P <0.05) are denoted by asterisks next to salinity or time (*P <0.05, ***P <0.001, ****P <0.0001). No significant interaction between factors was found, and post-hoc tests were therefore redundant.

**Figure 2:**
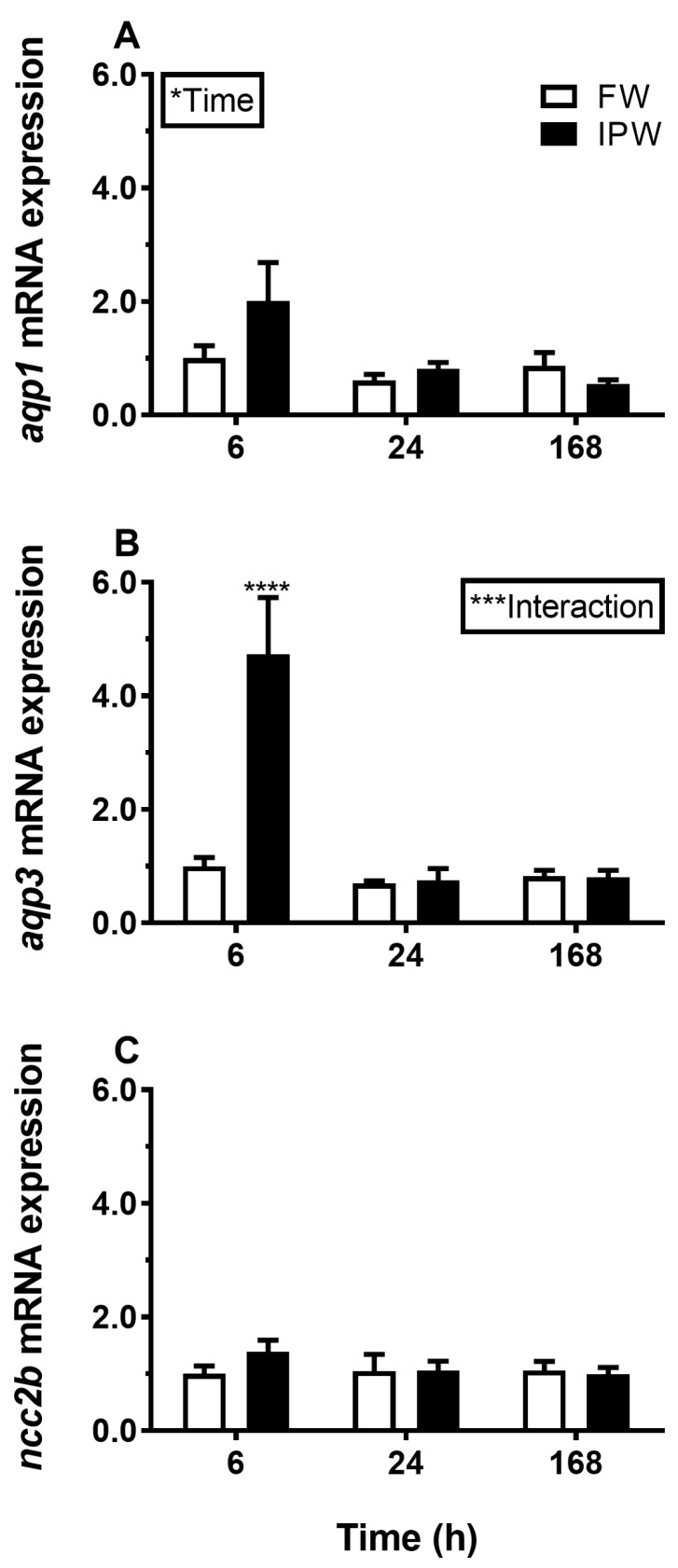
Effect of transfer from FW to 90% ion-poor water (IPW) on branchial *AQP1* (**A**), *AQP3* (**B**), and *NCC2b* (**C**) expression medaka at 6, 24, and 168 h timepoints. FW–FW sham transfer (open bars); FW–IPW transfer (solid bars). The level of each target was normalized to the geometric mean of the normalization genes *EF1a*, *bact*, and *RPL7* and presented relative to the control group at 6 h. Data are presented as mean ± standard error of the mean (n = 7–8). Statistically significant factor effects (two-way ANOVA, *P* < 0.05) are denoted by asterisks (*P <0.05). When the interaction between the time and salinity factor was significant (***P <0.001), asterisks were placed above the IPW group indicating difference from the time-matched control (Sidak multiple comparisons test, ****P <0.0001).

Transfer from FW to SW induced a significant and lasting overall decrease in *AQP3* mRNA in the gill compared with the FW–FW sham treatment (Figure 1B). By comparison, *AQP1* was not significantly affected by salinity (Figure 1A), and *NCC2b* responded by an overall decline in response to SW (Figure 1C). Exposure to IPW induced an initial increase in *AQP3* mRNA at 6 h followed by a return to basal FW levels (Figure 2B). *AQP1* and *NCC2b* mRNA levels were not significantly affected by transfer to IPW (Figure 2A,C).

### 3.2. Western Blotting and Aquaporin Protein Levels

**Figure 3:**
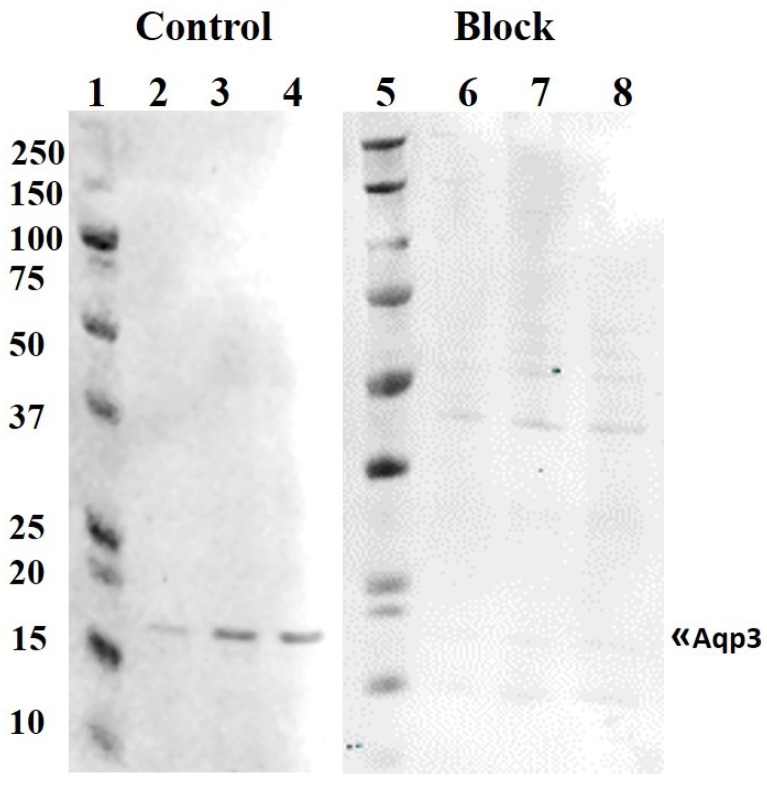
Western blot of FW medaka gill protein probed with an antibody against medaka AQP3. Lanes 2–4 were probed with the AQP3 antibody; lanes 5–8 were probed with the antibody after neutralization with 400x molar excess of the antigenic peptide. Lanes 1 and 5 contained a molecular mass marker (Protein Plus, BIO-RAD, Hercules, CA); lanes 2–4 and 6–8 contained 5, 10, and 15 μg of total loaded protein, respectively. A single immunoreactive band at ~18 kDa was detected, which was not present after antibody neutralization.

**Figure 4:**
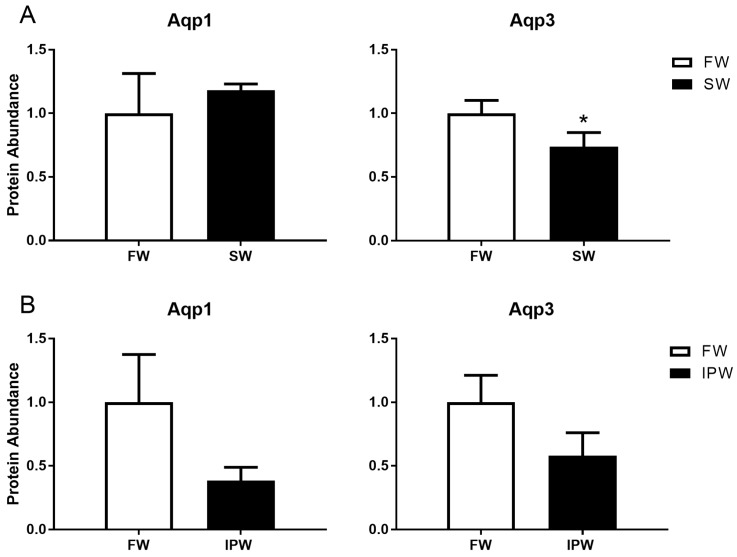
Western blot analysis of AQP1 (left) and AQP3 (right) protein levels in medaka gills 7 days after transfer from FW (open bars) to (**A**) SW (solid bars) or (**B**) IPW (solid bars). Immunoreactive AQP bands were analyzed and normalized to the level of β-actin in each sample. Values are presented relative to the FW control group. Statistically significant effects (two-tailed Student’s t-tests, *P* <0.05) are denoted by asterisks. Data are presented as mean ± standard error of the mean (n = 5–6).

An antibody was raised against medaka AQP3. In western blot analysis, the antibody recognized one major immunoreactive band with an apparent molecular weight of 18 kDa, which is lower than the predicted 33 kDa for medaka AQP3 (Figure 3). The 18 kDa band disappeared when the antibody was neutralized with excess antigenic peptide. A comparison of gill samples from 7-day acclimated FW and SW medaka showed no significant differences in AQP1 abundance, whereas AQP3 protein levels were lower in SW compared with those in FW (Figure 4A). Transfer to IPW for 7 days did not induce any significant change in the protein expression of either AQP1 or AQP3 compared with those in FW (Figure 4B).

### 3.3. Branchial Aquaporin Localization

**Figure 5:**
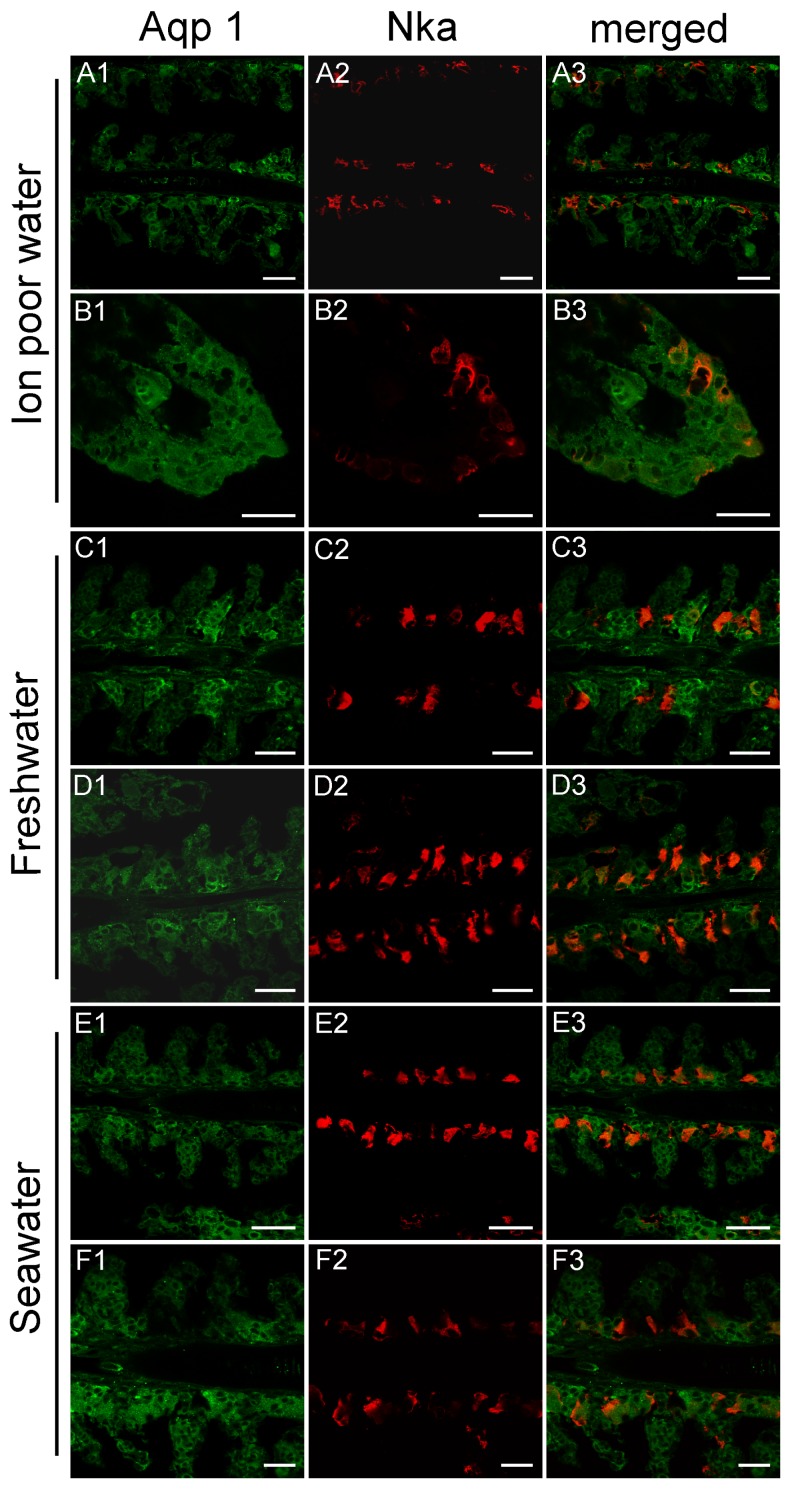
Representative confocal images of sagittal sections of medaka gills showing immunoreactivity against medaka AQP1 (green), the Na^+^,K^+^-ATPase alpha subunit (red), and merged. Rows A and B are from IPW fish, C and D are from FW fish, and E and F are from SW fish. Scale bar = 20 μm. For orientation see Figure 9A.

**Figure 6:**
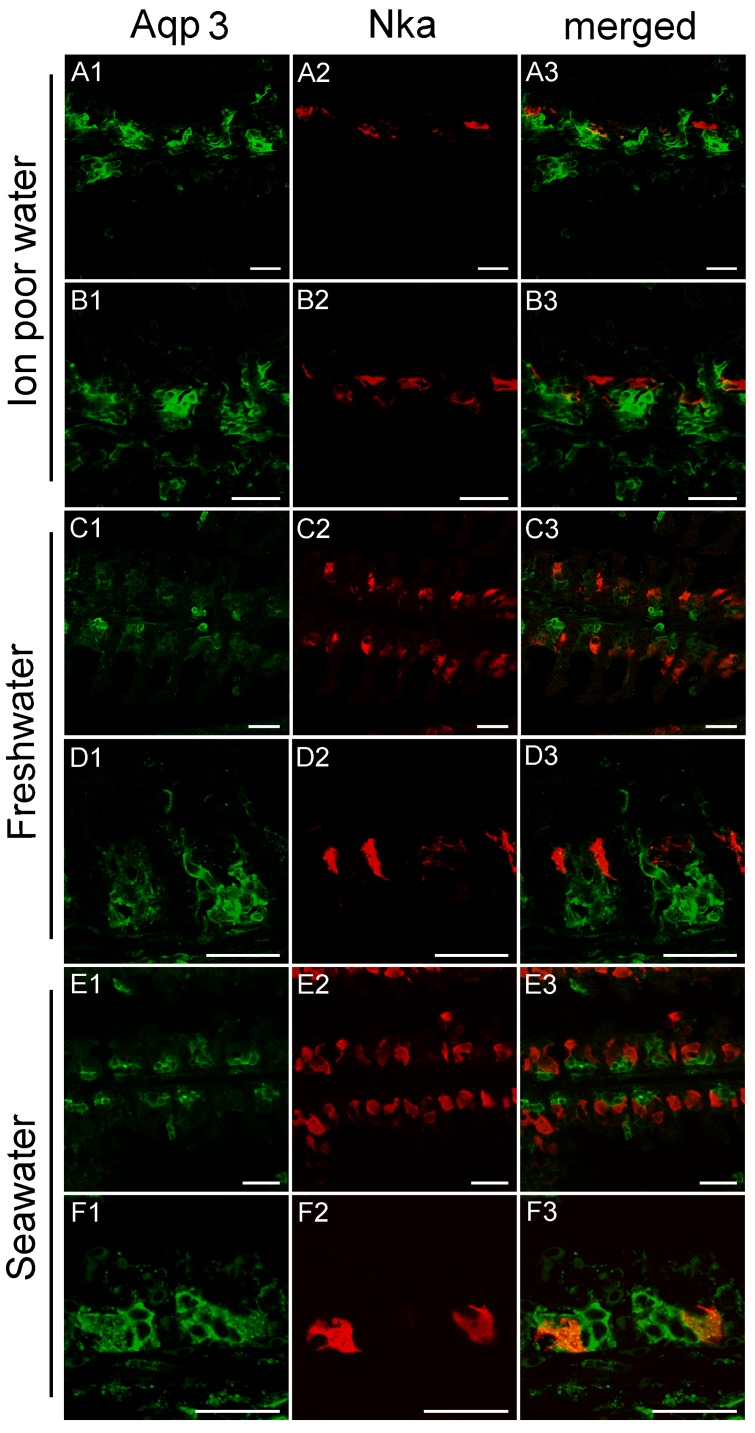
Representative confocal images of sagittal sections of medaka gills showing immunoreactivity against medaka AQP3 (green), the Na^+^,K^+^-ATPase alpha subunit (red), and merged. Rows A and B are from IPW fish, C and D are from FW fish, and E and F are from SW fish. Scale bar = 20 μm. For orientation see Figure 9A.

**Figure 7:**
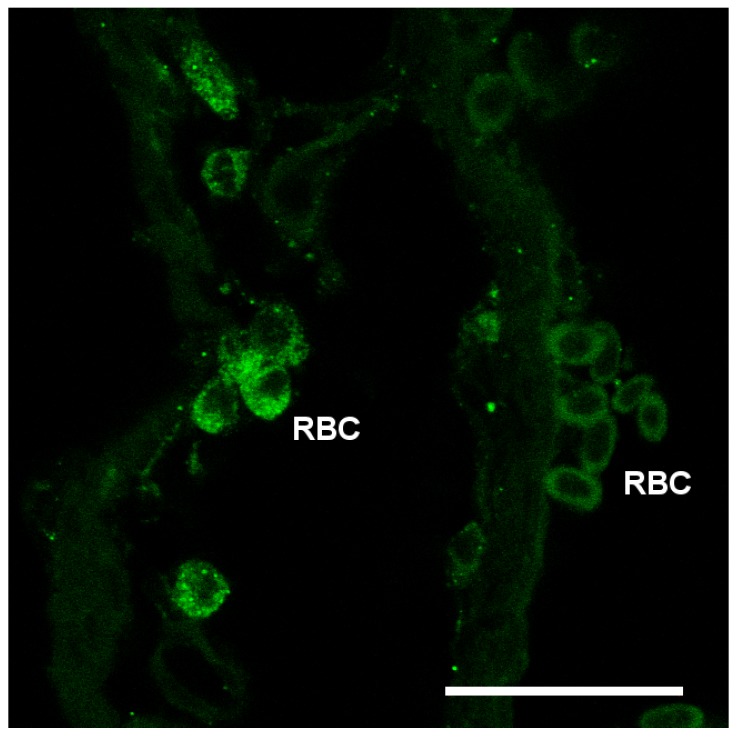
Confocal image of red blood cells (RBC) showing immunoreactivity against medaka AQP1 (green). Scale bar = 20 μm.

**Figure 8:**
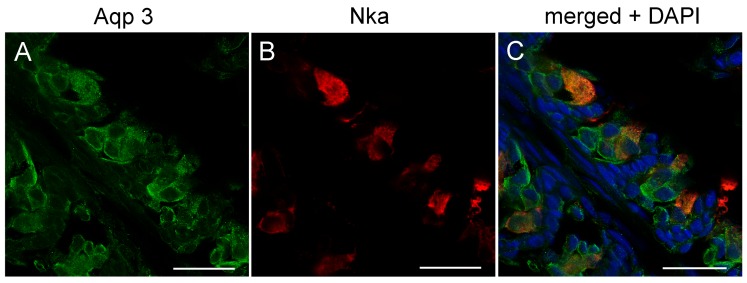
Confocal image of sagittal sections of FW medaka gills showing immunoreactivity against medaka (**A**) AQP3 (green), (**B**) Na^+^,K^+^-ATPase (red), and (**C**)merged channels overlaid with nuclear staining (blue 4’,6-diamidino-2-phenylindole (DAPI)). Scale bar = 20 μm.

**Figure 9:**
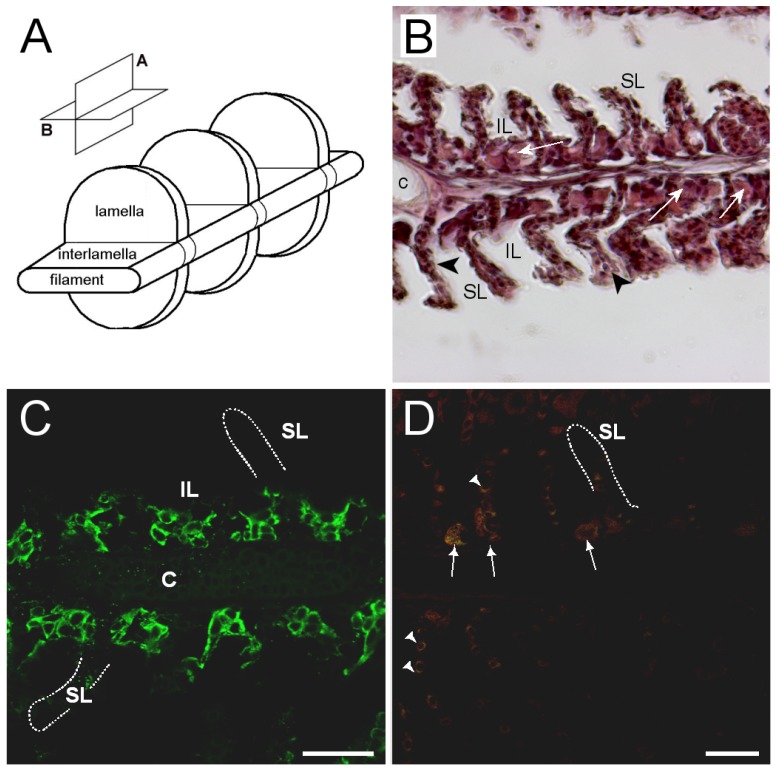
(**A**) three-dimensional (3D) diagram of gill filament morphology with orientation planes; (**B**) bright field image of sagittal gill section (plane A) of hematoxylin–eosin-stained gill filament; (**C**) representative confocal image of sagittal section from FW medaka gills showing immunoreactivity against medaka AQP3 (green)); and (**D**) negative control sagittal gill section incubated without primary antibodies. Abbreviations: SL = secondary lamella; IL = interlamellar space; and c = cartilage. Arrows point to ionocytes, and arrowheads point to red blood cells showing weak autofluorescence in (**D**). The outlines drawn in (**C**) and (**D**) show the contour of secondary lamellae for orientation. Scale bars = 20 μm.

Nka immunoreactivity (NKIR), which was used as a marker of branchial ionocytes appeared at high intensity in ionocytes along the gill filaments and was rarely observed in the lamellar epithelium (Figure 5 and Figure 6). NKIR cells were larger in the SW- than in the FW-acclimated medaka. AQP1 immunoreactivity in the gill was generally weak in IPW, FW, and SW (Figure 5) and was localized primarily in the periphery of cells in the interlamellar area of the filament. There was no apparent overlap between AQP1 immunoreactivity and NKIR, and the intensity was unaffected by environmental salinity. AQP1 was predominately localized in epithelial cells adjacent to NKIR cells on the filament. In addition, AQP1 immunoreactivity was observed in the membrane of red blood cells in the lamellae (Figure 7). AQP3 immunostaining was generally much stronger than AQP1 immunoreactivity and found in interlamellar space in the filament, that includes NKIR cells (Figure 6 and Figure 8). AQP3 staining appeared strongest in the deeper cell layers of the filament and was circumferential with nuclei unstained and centered in the middle (Figure 6). In Figure 8, nuclei were stained with DAPI, and there was clearly no overlap with the localization of AQP3. AQP3 staining appeared similar in intensity irrespective of salinity (Figure 6). In gill sections incubated without a primary antibody, there was a very weak autofluorescence in ionocytes, and red blood cells at both the green and red laser lines (Figure 9B).

### 3.4. Hormonal Effects on AQP Expression Ex Vivo

**Figure 10:**
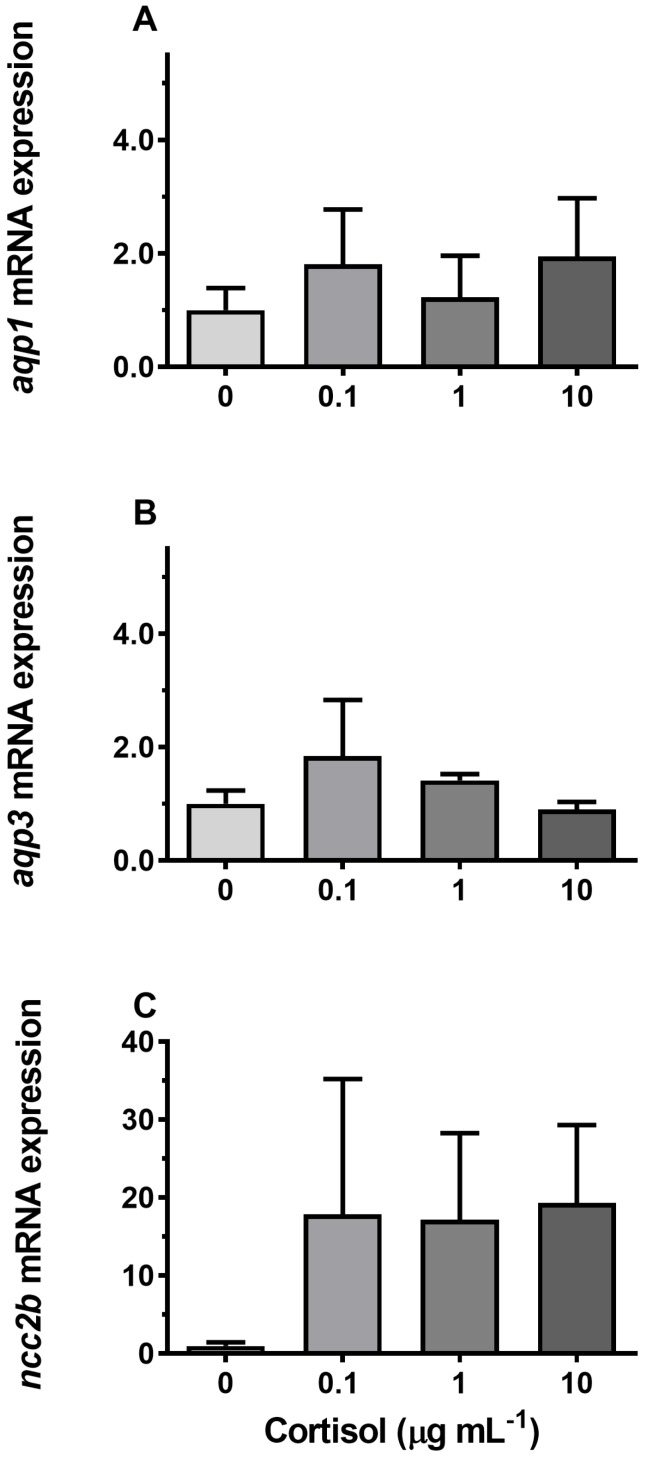
(**A**–**C**) Ex vivo dose–response of cortisol (0, 0.1, 1, and 10 μg mL^−1^) on *AQP1*, *AQP3*, and *NCC2b* expression in FW medaka gills. Gill explants were incubated for 18 h at room temperature. Expression of each target was normalized to the geometric mean of the three normalization genes: *EF1a*, *bact*, and *RPL7* and presented relative to the control. Data are presented as mean ± standard error of the mean (n = 4–6). No significant effects were observed (one-way ANOVA, *P* <0.05).

**Figure 11:**
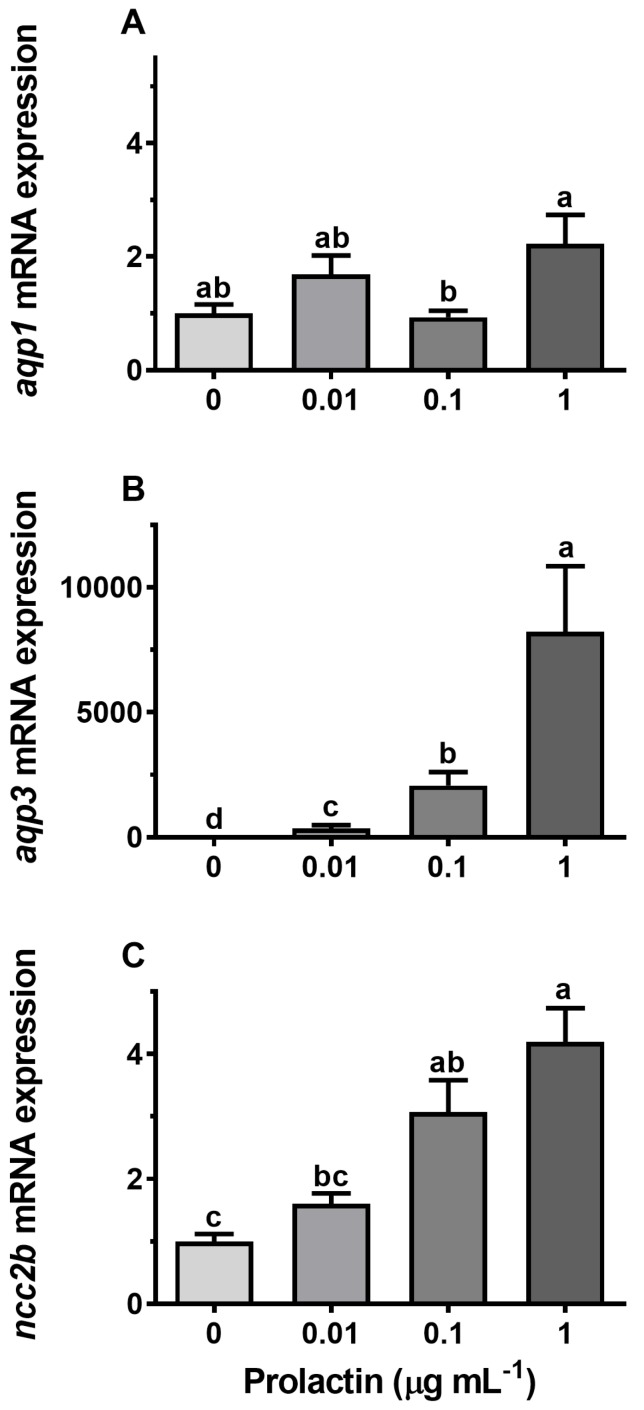
(**A**–**C**) Ex vivo dose–response of prolactin (PRL, 0.01, 0.1, and 1 μg mL^−1^) on *AQP1*, *AQP3*, and *NCC2b* expression in FW medaka gills. Gill explants were incubated for 18 h at room temperature. Expression of each target was normalized to the geometric mean of the three normalization genes: *EF1a*, *bact*, and *RPL7* and presented relative to the control. Data are presented as mean ± standard error of the mean (n = 7–8). Bars sharing a letter are not statistically different (one-way ANOVA, *P* <0.05).

**Figure 12:**
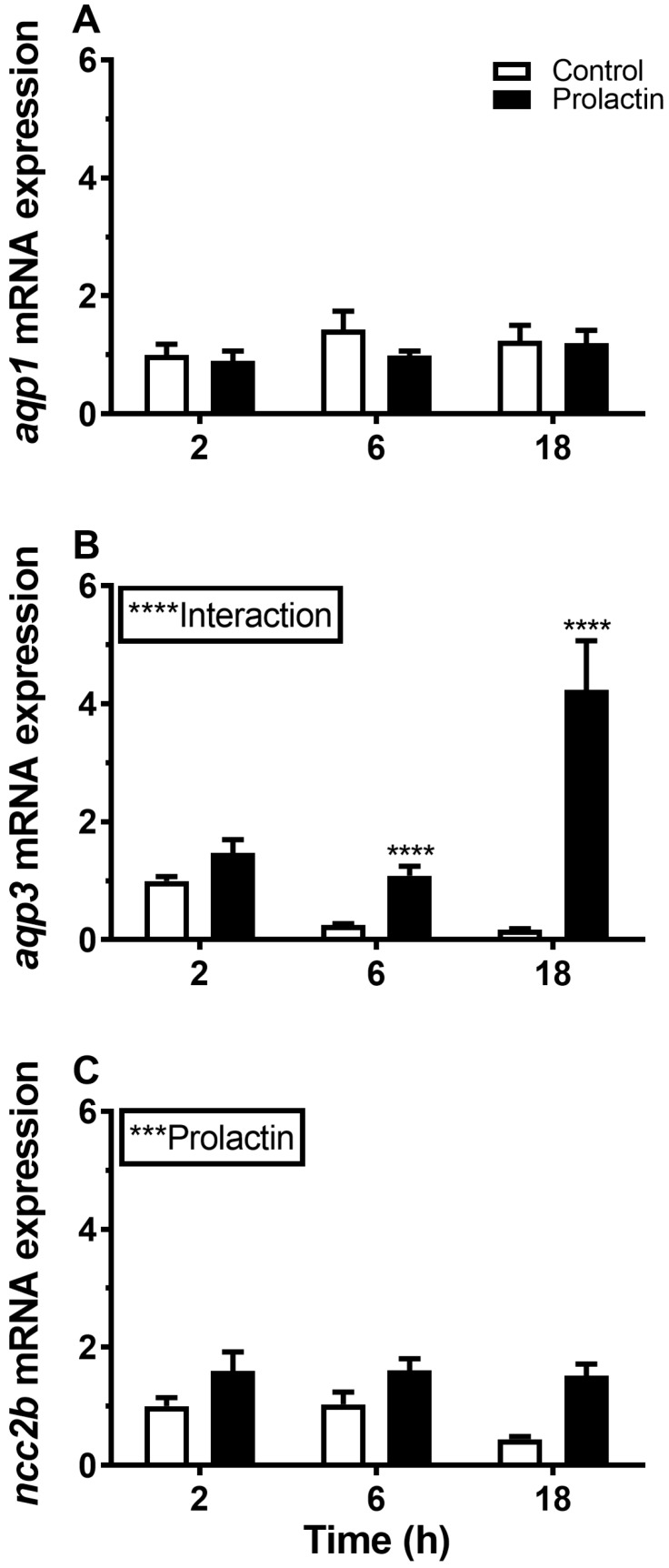
(**A**–**C**) Ex vivo time-course effect of ovine PRL (1 μg mL^−1^) on *AQP1*, *AQP3*, and *NCC2b* expression in FW medaka gills. Gill explants were incubated for 2, 6, and 18 h at room temperature. Control incubation (open bars); PRL incubation (solid bars). Expression of each target was normalized to the geometric mean of the three normalization genes: *EF1a*, *bact*, and *RPL7* and presented relative to the control. Data are presented as mean ± standard error of the mean (n = 7–8). Statistically significant factor effects (two-way ANOVA) are denoted by asterisks next to PRL (***P <0.001). When the interaction between the time and prolactin factor was significant, ****P <0.0001), asterisks were placed above the PRL group to indicate difference from the time-matched control (Sidak multiple comparisons test, ****P <0.0001).

**Figure 13:**
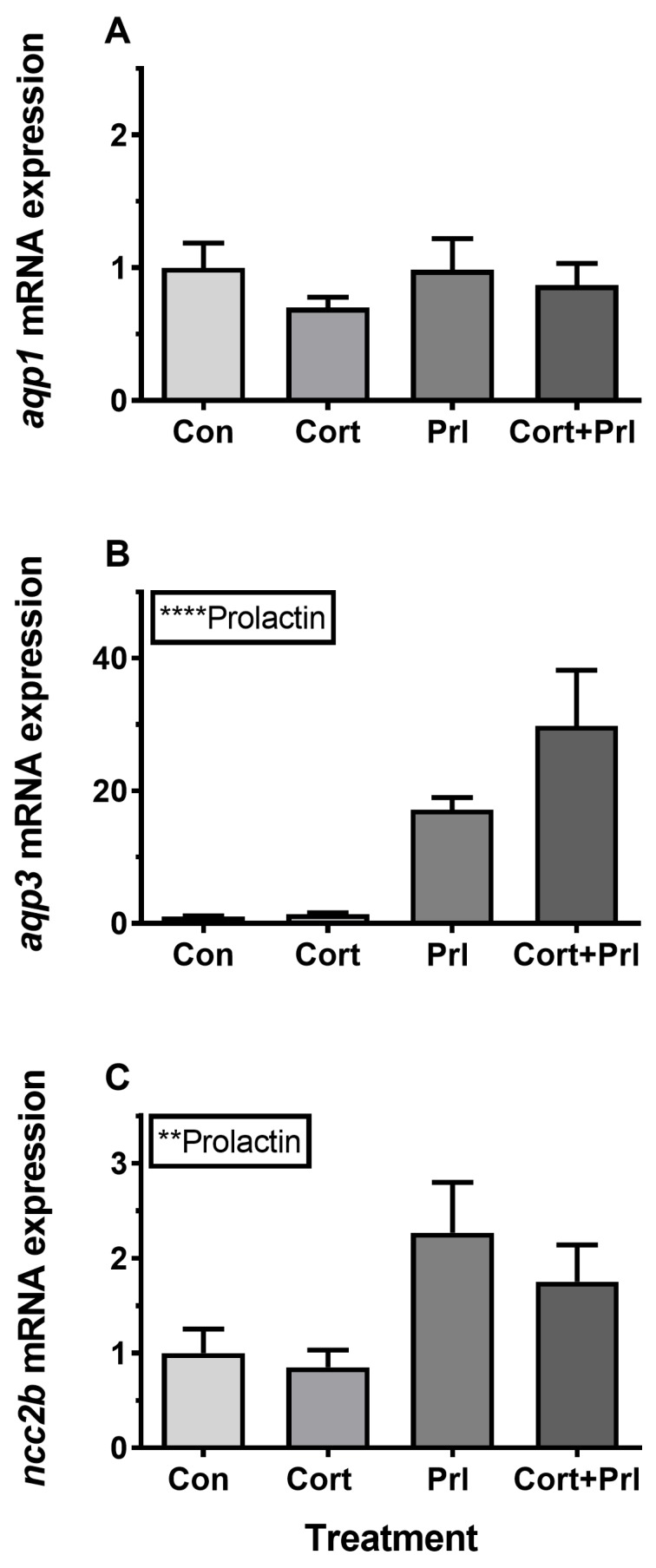
(**A**–**C**) Ex vivo effect of cortisol (10 μg mL^−1^), ovine PRL (1 μg mL^-1^), and their combination on *AQP1*, *AQP3*, and *NCC2b* expression in FW medaka gills. Gill explants were incubated for 18 h at room temperature. Expression of each target was normalized to the geometric mean of the three normalization genes: *EF1a*, *bact*, and *RPL7.* Data are presented as mean ± standard error of the mean (n = 7–8). Statistically significant overall effects of prolactin (two-way ANOVA, *P* <0.05) are denoted by asterisks: **P <0.01, ****P <0.0001. No significant interaction between cortisol and prolactin factor was found, and post-hoc tests were therefore redundant.

Incubation of gill explants for 18 h with cortisol had no significant effect on *AQP1, AQP3,* or *NCC2b* mRNA levels at any of the doses tested when compared with the control (Figure 10). Incubation with ovine PRL induced dose-dependent increases in *AQP3* and *NCC2b* mRNA levels and no overall effect on *AQP1* mRNA levels (Figure 11). The effect of PRL was further investigated in a time-course experiment with 2, 6, and 18 h of incubation. Stimulation of *AQP3* was significant after 6 h of incubation, while control *AQP3* expression decreased with time (Figure 12B). There was no effect of PRL or time on *AQP1* levels (Figure 12A), whereas there was an overall stimulatory effect of PRL on *NCC2b* (Figure 12C). To test for interactive effects of cortisol and PRL, a combined incubation with the two hormones was conducted. This experiment confirmed the overall stimulatory effect of PRL on *AQP3* and *NCC2b* expression, while cortisol had no significant effect alone nor any interactive effect with PRL (Figure 13B,C). *AQP1* expression was statistically unaffected by any of the hormone treatments (Figure 13A).

## 4. Discussion

Japanese medaka is a euryhaline fish, which tolerates experimental transfers between different salinities, such as FW and SW and in the present study, IPW. Previous studies have shown that ion-osmotic homeostasis is reestablished within a few days after transfer [32,33]. It therefore serves as a good model species to study the plasticity of osmoregulatory organs. The present study investigated events related to water balance in the gill and focused on the response of two aquaporin paralogs expressed in whole gill: AQP1 and AQP3. We demonstrated a differential response of *AQP1* and *AQP3* when exposed to changing salinity and further investigated how the response may be mediated by osmoregulatory hormones. AQP3 mRNA and protein is stimulated in hypo-osmotic environments, a response which is possibly mediated by PRL, whereas AQP1 is constitutively expressed at low levels and unaffected by both salinity and the hormones PRL and cortisol.

### 4.1. Salinity Response of Branchial Aquaporins

The two investigated aquaporins, *AQP1* and *AQP3* were both expressed in whole gill tissue, which is in accordance with Madsen et al. [33]. Judging from the Ct-values of individual samples in our qPCR analyses (*AQP3*: 22–24, *AQP1*: 28–30), *AQP3* is overall expressed at a much higher transcript level than *AQP1*. Furthermore, *AQP3* levels were consistently down-regulated when fish were acclimated from FW to SW and with an initial increase (6 h) after transfer from FW to IPW. The decreased transcript level in SW was corroborated by a decrease in AQP3 protein, while the level remained constant 7 days after transfer to IPW. Overall, this suggests that AQP3 serves an important homeostatic function in the gill in response to osmotic changes, in particular during acclimation to hypo-osmotic conditions. By contrast, AQP1 mRNA and protein were unaffected by the present osmotic manipulations, suggesting that this paralog may serve a generic function independent of the osmotic environment. Thus, a differential regulation of the two aquaporins occurs in the gill in response to changes in the osmotic environment. The *NCC2b* cotransporter gene was measured as a marker of FW-type NCC ionocytes. NCC2 is essential for NaCl homeostasis in hypo-osmotic environments by facilitating apical uptake of Na^+^ and Cl^−^ [39,40,41,42,43], and as expected we found that SW transfer was accompanied by a lasting decrease in *NCC2b* expression. Transfer to IPW, however, did not further stimulate *NCC2b* expression, suggesting that this transporter function is not a bottleneck in ion absorption under the present ion-poor conditions.

Previous papers have investigated the branchial expression of *AQP3* mRNA in different teleost species, and there is a developing consensus that expression of this aquaporin is lower in SW than in FW (European eel: [44,45]; Japanese eel: [46]; European seabass: [47,48]; tilapia: [27]; river pufferfish: [49]; silver seabream: [50]; Atlantic salmon: [51]; marine medaka: [52]; and killifish: [53]). A report on sockeye salmon, however, showed up-regulation of branchial *AQP3* in SW-acclimated parr, while it was down-regulated in smolts [54]. Branchial *AQP1* expression has also been investigated in relation to salinity in a few teleosts, but variable effects of salinity have been reported. In black porgy [55] and in marine medaka [52], a higher gill *AQP1* level was found in FW than in SW, whereas no salinity effect was found in seabass [47], river pufferfish [49], and climbing perch [56], the latter being in accordance with the present study in Japanese medaka. In Atlantic salmon, two related *AQP1* paralogs are expressed in gill tissue, *AQP1aa* and *AQP1ab*, which are inversely regulated by salinity [57], thus adding more diversity to the regulation of this aquaporin.

### 4.2. Immunolocalization and Physiological Function of Branchial Aquaporins

The cellular localization of the two aquaporin paralogs was investigated by immunofluorescence confocal microscopy. Our data showed a widespread immunoreactivity of AQP3 in cells in the interlamellar region of the filament, apparently in the periphery (membrane) of epithelial cells, as well as co-localizing with NKIR in ionocytes. This staining intensity was relatively weak and more scattered in ionocytes than other cell types, probably due to its localization in the basolateral tubular network. As reviewed by Madsen et al. [20], previous investigations have emphasized that AQP3 is localized in filament chloride cells co-localizing with Nka in European eel [45], Japanese eel [46], tilapia [58], rainbow wrasse [59], and killifish [53]. Furthermore, electron microscopy studies by Lignot et al. [45] and Watanabe et al. [58] demonstrated that this aquaporin was localized in the basolateral membrane network and not in the apical area facing the surrounding water. However, upon closer inspection of these studies, AQP3 is also present in additional cells in the interlamellar region of the filament, named accessory cells in the study of Brunelli et al. [59]. Tse et al. [46] found that *AQP3* is expressed in both chloride cells and pavement cells isolated from eel, and transcript levels decreased upon SW acclimation in both cell types. A study in killifish [53] reported AQP3 immunoreactivity to be present in pillar cells on the lamellae, which no other studies have confirmed, including the present study. Interestingly, a recent study in tilapia [27] reported AQP3 immunoreactivity in various epithelial cells of the gill, including ionocytes, mucous cells and pavement cells. The web-like mosaic of peripheral staining in interlamellar cells’ staining pattern in their study closely resembles what we presently observed in Japanese medaka. These cells may be of different types, such as accessory cells and/or immature ionocytes. Thus, despite possible species differences, it seems that AQP3 expression is not strictly confined to ionocytes but appears more widespread and may be important in other filament cell types.

Characteristically, AQP3 immunoreactivity appeared below the epithelial surface in cells deeper in the filament. The intensity of AQP3 immunoreactivity appeared quantitatively similar irrespective of salinity, thus not confirming the semi-quantitative western blot data showing slightly decreased whole gill AQP3 protein expression in SW fish. Based on the present localization pattern, it seems reasonable to conclude that AQP3 is functionally involved in epithelial cell volume control. Apical immunoreactivity in the epithelial surface facing the water was generally not observed. This makes sense, because steep osmotic gradients across the apical membrane of surface epithelia exist in both FW and SW, and low apical water permeability is important to keep transepithelial water fluxes to a minimum. Meanwhile, basolateral AQPs could support rapid cell volume adjustments coupled to the serosal compartment. It should also be kept in mind that AQP3 is classified as an aquaglyceroporin, meaning that it may facilitate transport of other small molecules, such as glycerol, which may have an important role in metabolism and the hydration status of these cells. In mammals, it is well established that AQP3 is expressed in the skin, where it is essential for epidermal glycerol transport and consequently hydration status [60].

The present data overall show very weak AQP1 immunoreactivity in the gill, matching the lower mRNA level of this paralog. The staining pattern resembles that of AQP3, except that AQP1 does not seem to be present in NKIR ionocytes. Furthermore, AQP1 immunoreactivity is unaffected by salinity, suggesting that the paralogs have a generic function. As expected, AQP1 is also expressed in red blood cells, which agrees with the consensus in mammals [20,61]. When analyzing whole tissue mRNA levels, the expression in (nucleated) red blood cells may have masked subtle changes in mRNA expression within the epithelial cells in response to salinity. However, it is not possible to avoid this problem when analyzing whole tissue expression. The present protein data (western and immunofluorescence) support the stability of AQP1 expression. Localization of branchial AQP1 has only been investigated in a few studies. Using a homologous antibody, Cerdá and Finn [62] detected AQP1 along the surface of the gill lamellae in zebrafish, whereas Brunelli et al. [59] found strong reactivity in chloride cells in the rainbow wrasse using a heterologous rat AQP1 antibody. Preliminary data from Atlantic salmon also show AQP1 immunoreactivity in chloride cells using a homologous antibody (unpublished observations: Madsen, S.S.). Kwong et al. [63] localized AQP1 basolaterally in ionocytes of the yolk sac of zebrafish larvae, where it was assigned a role in volume regulation. Thus, there seems to be species differences in localization of AQP1, and unfortunately, choice and quality of antibody may add to this divergence. AQP1 is also suspected of playing a role in CO_2_ (and NH_3_) transport [64,65]; the former may be of functional importance in red blood cells and the latter may contribute to ammonia excretion across the gill surface [56].

### 4.3. Endocrine Regulation of Branchial Aquaporins

Surprisingly little attention has been given to the endocrine regulation of aquaporin expression in any vertebrate tissue or cell type. Whereas many molecular details about the classic vasopressin effect on AQP2 in the mammalian nephrons [66] are known, other hormones and other AQPs remain largely unexplored. In fishes, the first study that addressed the endocrine regulation of any AQP was in European eel [67], where cortisol down-regulates *AQP3* mRNA in gills, thus mimicking the effect of SW-acclimation in this species. Due to the relatively small magnitude of the effect, the authors concluded that other factors might be involved in order to mediate the effect of hyper-osmotic exposure. Many years later, Breves et al. [27] followed up on this initial attempt to get insight into the endocrinology of branchial AQP3 regulation and demonstrated in a series of in vivo and ex vivo experiments that pituitary PRL is a potent stimulatory factor of branchial AQP3 expression at both the mRNA and protein level. They also found that cortisol antagonizes the action of PRL, which supports the data of Cutler et al. [67] in eel. At the same time, PRL may be the factor to fill in the gap in Cutler et al.’s study, because circulating PRL levels are known to be high in FW and low in SW, defining PRL as a FW hormone in teleosts [25]. Our data in medaka support the conclusion that PRL stimulates branchial *AQP3* expression in a dose- and time-dependent manner. As a control for the FW-acclimating action of PRL, we confirmed that the transcript level of the *NCC2* gene was also stimulated by the hormone, which is in agreement with Breves et al.’s [28,68] observation in tilapia and zebrafish. *AQP3* has been linked to the osmosensing ability of Mozambique tilapia pituitary PRL secreting cells and their release of PRL in hypo-osmotic environments, further connecting this hormone with hypo-osmotic acclimation [69]. In comparison with *AQP3*, *AQP1* expression was unaffected by PRL, which is congruent with the lack of effect of the osmotic environment. To our knowledge, no other studies have investigated hormonal effects on gill AQP1 in any teleost. Cortisol, the main corticosteroid in teleost fish, did not have a significant effect on *AQP3* expression either alone or in combination with PRL. The effect of PRL was also not modulated by the presence of cortisol in ex vivo hormone experiments, which is contrary to findings in European eel [67] and in tilapia [68].

## 5. Conclusions

This study has increased our understanding of the localization and of AQP1 and AQP3 within a euryhaline fish gill. There is a partial overlap in the cellular localization of these two paralogs in the gill epithelium, suggesting that they both play roles in cellular volume homeostasis. They are however, differentially regulated in response to environmental salinity and hormonal manipulation. AQP1 is stably expressed irrespective of salinity and hormone manipulation, whereas AQP3 mRNA and protein respond dynamically and inversely to environmental salinity and are stimulated by PRL. Transfer from FW to ion-poor conditions does not further affect AQP3 expression, suggesting that cell volume homeostasis is not further challenged by this manipulation.
